# The role of mTOR signalling in the regulation of skeletal muscle mass in a rodent model of resistance exercise

**DOI:** 10.1038/srep31142

**Published:** 2016-08-09

**Authors:** Riki Ogasawara, Satoshi Fujita, Troy A. Hornberger, Yu Kitaoka, Yuhei Makanae, Koichi Nakazato, Ishii Naokata

**Affiliations:** 1 Department of Life Science and Applied Chemistry, Nagoya Institute of Technology, Nagoya, Japan; 2 Department of Life Sciences, The University of Tokyo, Tokyo, Japan; 3 Department of Sport and Health Science, Ritsumeikan University, Kusatsu, Japan; 4 Department of Comparative Biosciences, School of Veterinary Medicine, University of Wisconsin-Madison, Madison, Wisconsin, USA; 5 Department of exercise physiology, Nippon Sport Science University, Tokyo, Japan

## Abstract

Resistance exercise (RE) activates signalling by the mammalian target of rapamycin (mTOR), and it has been suggested that rapamycin-sensitive mTOR signalling controls RE-induced changes in protein synthesis, ribosome biogenesis, autophagy, and the expression of peroxisome proliferator gamma coactivator 1 alpha (PGC-1α). However, direct evidence to support the aforementioned relationships is lacking. Therefore, in this study, we investigated the role of rapamycin-sensitive mTOR in the RE-induced activation of muscle protein synthesis, ribosome biogenesis, PGC-1α expression and hypertrophy. The results indicated that the inhibition of rapamycin-sensitive mTOR could prevent the induction of ribosome biogenesis by RE, but it only partially inhibited the activation of muscle protein synthesis. Likewise, the inhibition of rapamycin-sensitive mTOR only partially blocked the hypertrophic effects of chronic RE. Furthermore, both acute and chronic RE promoted an increase in PGC-1α expression and these alterations were not affected by the inhibition of rapamycin-sensitive mTOR. Combined, the results from this study not only establish that rapamycin-sensitive mTOR plays an important role in the RE-induced activation of protein synthesis and the induction of hypertrophy, but they also demonstrate that additional (rapamycin-sensitive mTOR-independent) mechanisms contribute to these fundamentally important events.

Protein metabolism plays a critical role in the regulation of skeletal muscle mass and recent studies have demonstrated that signalling by the mechanistic/mammalian target of rapamycin (mTOR) plays a central role in the regulation of both protein synthesis and protein degradation^1^,^2^. It is known that mTOR can be found in at least two multi-protein complexes called mTORC1 and mTORC2. The defining component of mTORC1 is a protein called Raptor, and it has been shown that a subset of mTORC1-dependent, but not mTORC2-dependent, signalling events are highly sensitive to inhibition by rapamycin^3^,^4^,^5^. For this reason, it has been widely assumed that mTORC1 is responsible for the rapamycin-sensitive and mTOR-dependent signalling events that regulate protein metabolism, yet recent studies have shown that this assumption may not be entirely correct^6^. Nonetheless, it is clear that rapamycin-sensitive mTOR signalling can regulate protein synthesis and autophagy and these effects are mediated, at least in part, through changes in the phosphorylation of downstream molecules such as p70S6K, 4E-BP1, and ULK1 1,2.

Consistent with its critical role in the regulation of protein metabolism, previous studies have shown that rapamycin-sensitive mTOR signalling is necessary for the hypertrophy that occurs in response to chronic mechanical overload^7^,^8^. Moreover, numerous studies have reported that rapamycin-sensitive mTOR is robustly activated for a relatively long duration (e.g. >24 h after exercise) after a single bout of RE^9^,^10^,^11^,^12^. Thus, it has been widely suggested that rapamycin-sensitive mTOR is the key signalling node through which RE induces hypertrophy. However, this hypothesis has not been fully tested in a model that mimics human RE. This is a critical point because the mode and pattern of muscle contraction, and the time course and the extent of adaptations that occur in response to chronic mechanical overload (via synergist ablation, SA) are quite different from RE. For example, SA increases muscle mass more than 50% within 2 weeks while RE takes a few months to increase muscle mass by 10%. Furthermore, a recent study which used a rodent model of RE reported that the inhibition of rapamycin-sensitive mTOR signalling only partially inhibits the increase in protein synthesis that is observed after RE^13^, indicating that, in contrast to SA, RE might induce muscle hypertrophy via both rapamycin-sensitive mTOR-dependent and -independent mechanisms.

In addition to its role in the regulation of protein metabolism, rapamycin-sensitive mTOR has also been shown to regulate mitochondria biogenesis via control of peroxisome proliferator gamma coactivator 1 alpha (PGC-1α) expression^14^. Increased PGC-1α expression occurs after endurance exercise via robust AMP-activated protein kinase (AMPK) activation^15^, and RE is known to increase PGC-1α expression^16^. Moreover, recent studies reported that the addition of RE or protein diet supplementation to endurance exercise results in amplified PGC-1α expression as compared with endurance exercise alone^17^,^18^. It has been proposed that these effects are at least partly due to rapamycin-sensitive mTOR activation; however, the role of rapamycin-sensitive mTOR in this process has not been directly addressed.

As described above, it has been widely suggested that rapamycin-sensitive mTOR signalling plays a key role in several of the adaptive responses that are observed following RE: however, the validity of these claims has not been fully investigated. Therefore, the purpose of this study was to define the role that rapamycin-sensitive mTOR plays in the RE-induced activation of muscle protein synthesis, ribosome biogenesis, PGC-1α expression and hypertrophy using a rodent model of resistance exercise.

## Results

### The effect of rapamycin on RE-induced Akt-rapamycin-sensitive mTOR signalling

**Figure 1:**
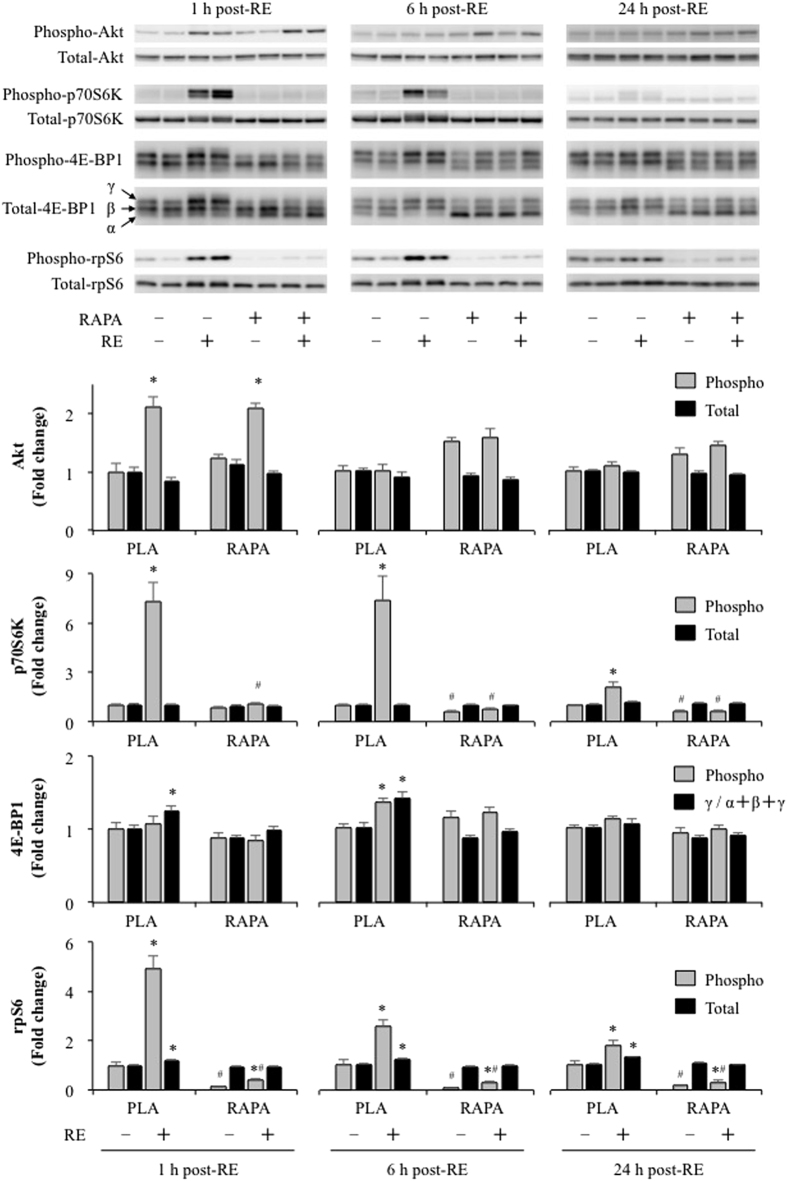
The effect of acute RE and rapamycin administration on the phosphorylation and total protein content of Akt, p70S6K, 4E-BP1, and rpS6. RE, resistance exercise; RAPA, rapamycin; PLA, placebo. Values in the graphs are means + SE. *P < 0.05 vs. no exercise control muscle in the same group; ^#^*P* < 0.05 vs. corresponding muscle in the placebo group.

Akt is known as effector of insulin/IGF-1 signalling and it can induce muscle hypertrophy through a pathway involving rapamycin-sensitive mTOR^19^. As shown in Fig. 1, the phosphorylation of Akt at Ser473 increased 1 h after RE independent of rapamycin administration while no significant differences were observed at 6 and 24 h post RE (Fig. 1). In contrast to the changes in Akt phosphorylation, rapamycin completely inhibited the RE-induced increase in p70S6K Thr389 phosphorylation at all time points. This is important because p70S6K Thr389 phosphorylation is a validated marker of rapamycin-sensitive mTOR signalling^7^, and thus, these results establish that the rapamycin treatment was fully effective at inhibiting rapamycin-sensitive mTOR signalling.

We next investigated other commonly used markers of rapamycin-sensitive mTOR signalling including 4E-BP1 and rpS6. Phosphorylation of 4E-BP1 at Thr37/46, which is known to be a direct target of rapamycin-sensitive mTOR^20^, increased only 6 h after RE and was rapamycin-sensitive. The active isoform ratio of 4E-BP1 (active γ-form / total α+β+γ), which is a marker of 4E-BP1 activity^21^,^22^, also increased 1 and 6 h after exercise and this effect was also completely inhibited by rapamycin. Moreover, RE also induced a significant increase in rpS6 Ser240/244 phosphorylation at all time points, and consistent with other studies^23^,^24^, this effect was not entirely blocked by rapamycin. Interestingly, RE also induced a small but significant increase total rpS6 at all time points and this effect was rapamycin-sensitive.

**Figure 2:**
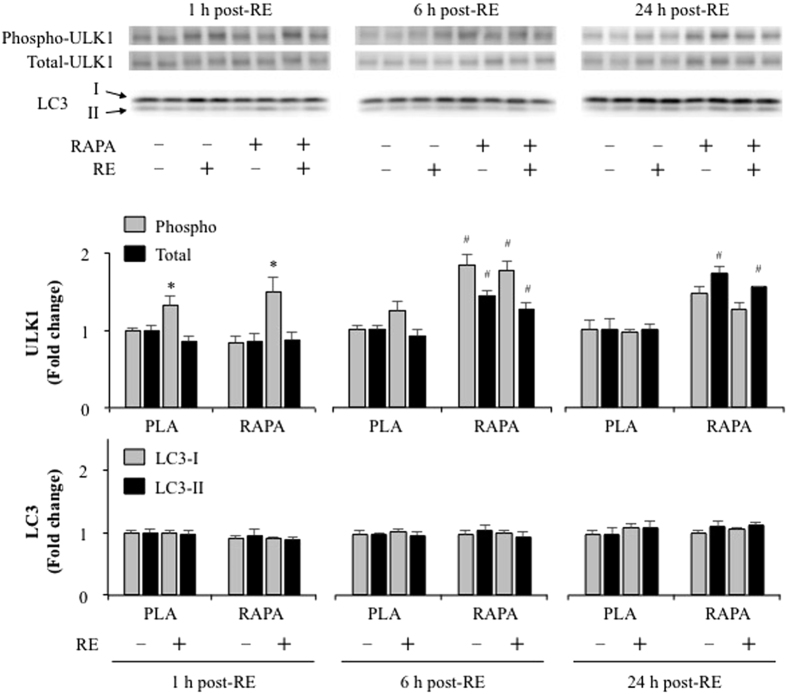
The effect of acute RE and rapamycin administration on the phosphorylation and total protein content of ULK1 and LC3. RE, resistance exercise; RAPA, rapamycin; PLA, placebo. Values in the graphs are means + SE. *P < 0.05 vs. no exercise control muscle in the same group; ^#^*P* < 0.05 vs. corresponding muscle in the placebo group.

ULK1 can activate autophagy and rapamycin-sensitive mTOR has been reported to inhibit ULK1 via phosphorylation of the Ser757 residue^25^. As shown in Fig. 2, the phosphorylation of ULK at Ser757 increased 1 h after RE independent of rapamycin administration, while no significant effect of RE was observed at 6 and 24 h post RE. In addition, no changes in LC3-I and -II proteins (autophagosome marker) were observed after RE and rapamycin administration. Combined, these results indicate that RE can increase ULK1 phosphorylation through an rapamycin-sensitive mTOR-independent process, but this event is not sufficient to alter LC3-based markers of autophagosome formation.

We further investigated MAPK signalling molecules, ERK1/2 and p38 MAPK, to determine whether there are off-target rapamycin effects. As shown in Fig. S2, the phosphorylation of ERK1/2 at Thr202/Tyr204 and p38 MAPK at Thr180/Tyr182 did not change after acute RE or rapamycin administration. Similarly, RE or rapamycin did not alter total ERK1/2 and p38 MAPK proteins.

### The effect of chronic rapamycin administration and RE on Akt-rapamycin-sensitive mTOR signalling

**Figure 3:**
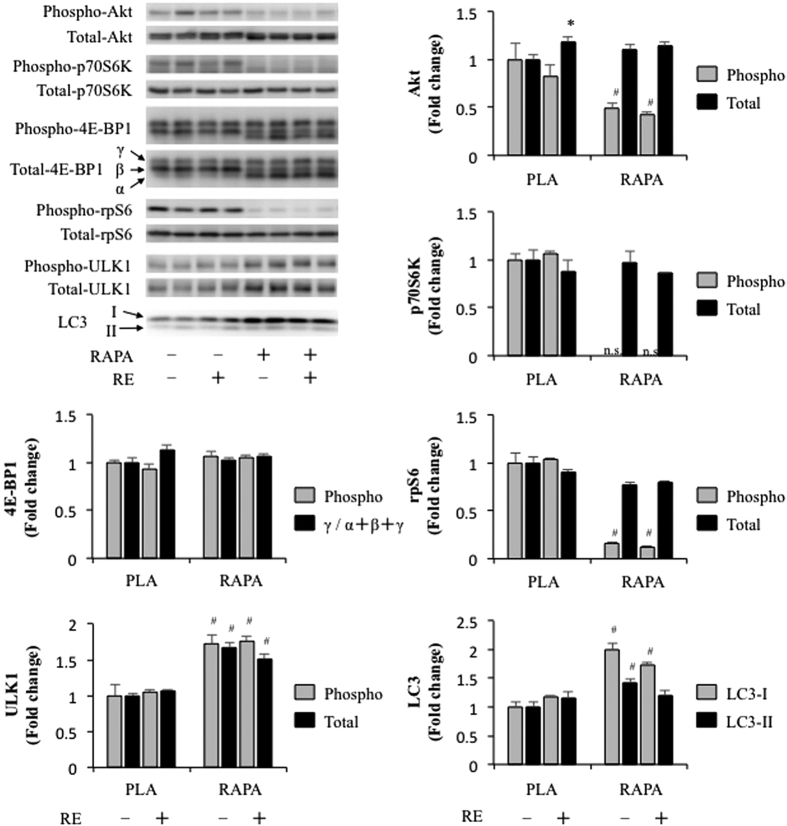
The effect of chronic RE and rapamycin administration on the phosphorylation and total protein content of molecules associated with RSmTOR signalling. RE, resistance exercise; RAPA, rapamycin; PLA, placebo. Values in the graphs are means + SE. *P < 0.05 vs. no exercise control muscle in the same group; ^#^*P* < 0.05 vs. corresponding muscle in the placebo group.

Chronic rapamycin administration (8 mg/kg rapamycin every other day for 4 weeks) has been reported to decrease basal phosphorylation of p70S6K, rpS6, and 4E-BP1 (increase in inactive α-form/total 4E-BP1) in skeletal muscle^26^, and similar results were observed in the present study (Fig. 3). Moreover, and in contrast to acute rapamycin administration, we observed that chronic rapamycin administration led to a decrease in basal Akt Ser473 phosphorylation (a marker of mTORC2 signalling). The decrease in Akt Ser473 phosphorylation is consistent with a previous study that administered 2 mg/kg/day of rapamycin for 4 weeks^27^, and therefore, lends support to the hypothesis that chronic rapamycin administration can inhibit both mTORC1 and mTORC2 in skeletal muscle^27^,^28^.

In contrast to downstream signalling molecules of rapamycin-sensitive mTOR that regulate protein synthesis, chronic rapamycin administration unexpectedly increased the phosphorylation and total content of ULK1 (Fig. 3), while the ratio of phosphorylated to total ULK1 was not changed. These results suggest that, at least in skeletal muscle, rapamycin-sensitive mTOR does not phosphorylate ULK1, but it may negatively control ULK1 expression and therefore the capacity of ULK1 signalling that leads to autophagy. Similarly, chronic rapamycin administration significantly increased LC3-I as well as LC3-II to an even greater extent (Fig. 3). It is known that immediately after translation, proLC3 is cleaved to inactive form, LC3-I 29. During autophagy, LC3-I is processed to the autophagosomal membrane-associated form, LC3-II, and the amount of LC3-II is correlated with the extent of autophagosome formation^29^. Thus, our results suggest that chronic inhibition of rapamycin-sensitive mTOR controls LC3 expression, but not necessarily autophagosome formation.

Basal phosphorylation of ERK1/2 at Thr202/Tyr204 and p38 MAPK at Thr180/Tyr182 were not changed after chronic rapamycin administration (Fig. S2). In contrast, chronic RE decreased basal phosphorylation of ERK1/2 at Thr202/Tyr204 but not p38 MAPK at Thr180/Tyr182 independent of rapamycin administration.

### The role of rapamycin-sensitive mTOR signalling in the regulation of RE-induced PGC-1α expression

**Figure 4:**
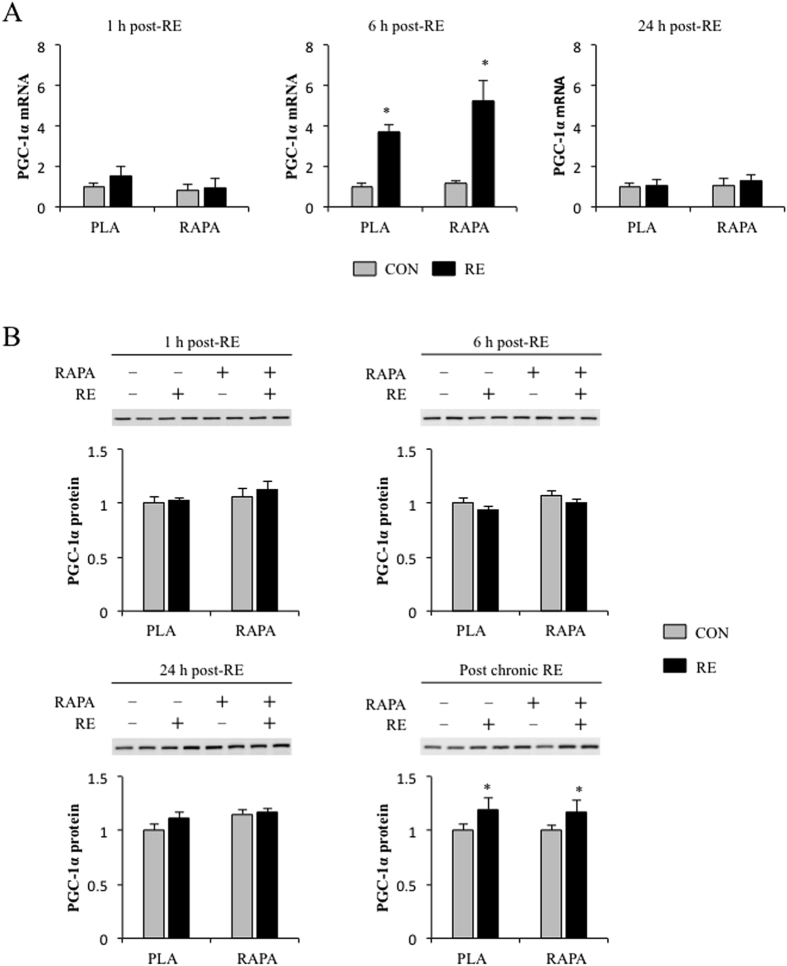
The effect of RE and rapamycin administration on PGC-1α mRNA (**A**) and protein (**B**). CON, control; RE, resistance exercise; PLA, placebo; RAPA, rapamycin. Values in the graphs are means + SE. *P < 0.05 vs. no exercise control muscle in the same group.

Rapamycin-sensitive mTOR has also been reported to regulate PGC-1α expression, which is a key regulator of mitochondria biogenesis^14^, and the expression of PGC-1α has been implicated in the control of skeletal muscle mass^30^,^31^. We found that RE potently increased the mRNA abundance of PGC-1α independent of rapamycin administration at 6 h after RE (Fig. 4A). However, no significant changes in PGC-1α protein levels were detected at any time point after acute RE. On the other hand, PGC-1α protein levels were significantly elevated after chronic RE, but rapamycin did not alter this response RE (Fig. 4B). Taken together, these results indicate that chronic RE induces an increase in PGC-1α and that this effect is mediated through an rapamycin-sensitive mTOR-independent mechanism.

### The role of rapamycin-sensitive mTOR signalling in the regulation of RE-induced ribosome biogenesis

**Figure 5:**
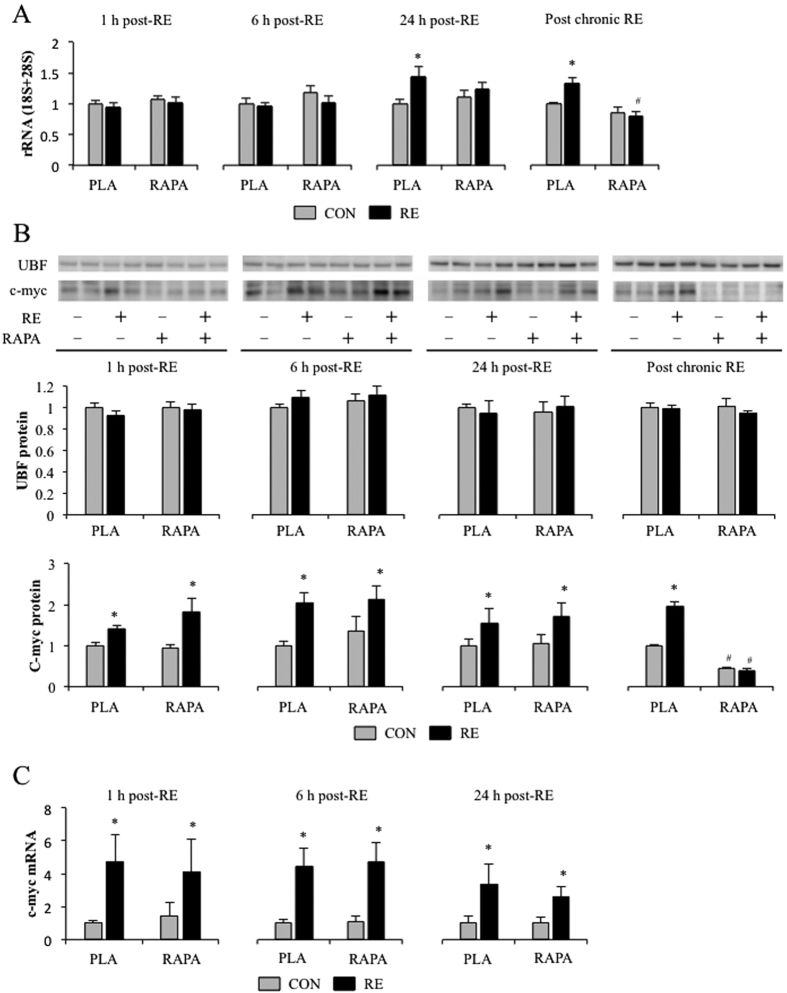
The effect of RE and rapamycin administration on rRNA (**A**), c-myc and UBF proteins (**B**), and c-myc mRNA (**C**). CON, control; RE, resistance exercise; PLA, placebo; RAPA, rapamycin. Values in graphs are means + SE. *P < 0.05 vs. no exercise control muscle in the same group; ^#^*P* < 0.05 vs. corresponding muscle in the placebo group.

Rapamycin-sensitive mTOR is known to stimulate not only translation initiation but also ribosome biogenesis^32^,^33^. Ribosomes act as the translation machinery, and thus their volume reflects the translational capacity of the cells. While enhanced translational efficiency, mainly via an increase in translation initiation, is considered to be important in enhancing muscle protein synthesis, ribosome biogenesis can also play a major regulatory role^32^. In the present study, RE did not alter rRNA content at 1 and 6 h after exercise, but significant increases in rRNA were observed 24 h after acute RE and chronic RE. These alterations were largely abolished by rapamycin (Fig. 5A). Thus, it can be concluded that both acture and chronic RE induce ribosome biogenesis through an rapamycin-sensitive mTOR-dependent pathway.

To examine possible mediators, we first looked at UBF because it is a transcription factor that induces rDNA transcription and it is recognized as a downstream regulator of rapamycin-sensitive mTOR^34^. However, neither acute nor chronic RE significantly altered UBF expression (Fig. 5B). Next, we looked at c-myc because ribosome biogenesis is known to be regulated by c-myc, and RE has been reported to promote an increase in c-myc expression^16^,^35^,^36^. Moreover, it has been suggested that c-myc expression might be regulated, at least in part, by rapamycin-sensitive mTOR^37^. Similar to previous studies, we found that both acute and chronic RE increased c-myc expression (Fig. 5B,C). Interestingly, rapamycin inhibited the increase in c-myc with chronic RE but not acute RE. Taken together, these data indicate that c-myc is not part of the rapamycin-sensitive mTOR pathway that drives acute RE-induced changes in ribosome biogenesis, however, c-myc might play role in the rapamycin-sensitive mTOR pathway that mediates ribosome biogenesis during chronic RE.

### The role of rapamycin-sensitive mTOR in RE-induced protein synthesis and muscle hypertrophy

**Figure 6:**
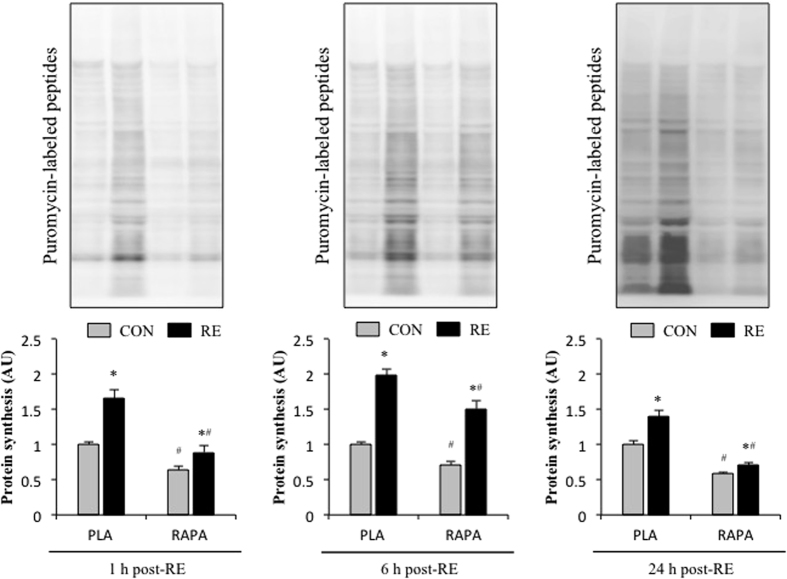
The effect of acute RE and rapamycin administration on muscle protein synthesis (puromycin-labeled peptides). CON, control; RE, resistance exercise; PLA, placebo; RAPA, rapamycin. Values are expressed relative to the no exercise placebo control group and presented as the means + SE. *P < 0.05 vs. no exercise control muscle in the same group; ^#^*P* < 0.05 vs. corresponding muscle in the placebo group.

Numerous studies have shown that RE induces an increase in muscle protein synthesis^38^,^39^, and it is thought that the increase in protein synthesis plays a central role in the concomitant hypertrophic response^40^. In accordance with previous studies^38^,^39^, we found that RE induced an increase in muscle protein synthesis at all of the time points that were analyzed (Fig. 6). Interestingly, however, rapamycin only partially inhibited the RE-induced increase in protein synthesis. Our observations are very similar to those recently reported by West *et al*.^13^, and therefore, provide further evidence in support the conclusion that both rapamycin-sensitive mTOR-dependent and -independent mechanisms contribute to the increase in protein synthesis that occurs after an acute bout of RE.

**Figure 7:**
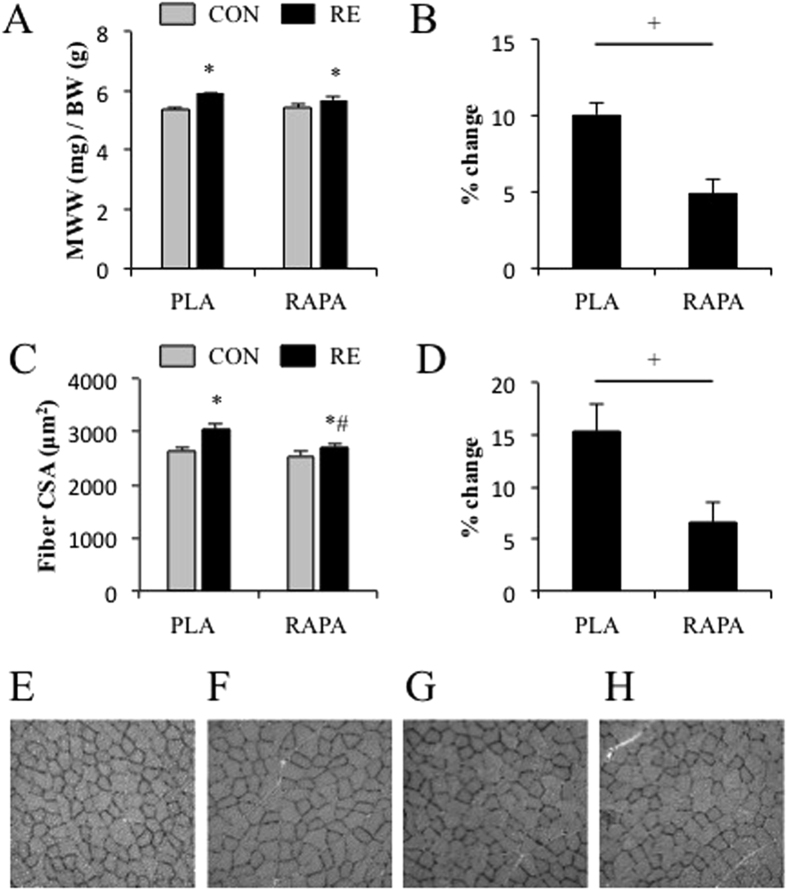
The effect of chronic RE and rapamycin administration on muscle wet weight, MWW (**A**,**B**) and fiber cross-sectional area, CSA (**C**,**D**). Representative images of PLA-CON (**E**), PLA-RE (**F**), PARA-CON (**G**), and RAPA-RE (**H**). CON, control; RE, resistance exercise; PLA, placebo; RAPA, rapamycin. Values are expressed relative to the no exercise placebo control group and presented as the means + SE. *P < 0.05 vs. no exercise control muscle in the same group; ^#^*P* < 0.05 vs. corresponding muscle in the placebo group; ^+^*P* < 0.05 between groups.

Our observation that an rapamycin-sensitive mTOR-independent mechanism contributes to the RE-induced increase in protein synthesis led us to the hypothesis that an rapamycin-sensitive mTOR-independent mechanism would also contribute to the hypertrophy that occurs in response to chronic RE. Thus, we next investigated the role of rapamycin-sensitive mTOR in chronic RE-induced muscle hypertrophy. The results show that chronic RE increased muscle wet weight (MWW) and fiber CSA in both placebo and rapamycin groups although the magnitude of increase in MWW and fiber CSA was significantly greater in the placebo group (Fig. 7).

## Discussion

RE is known to activate rapamycin-sensitive mTOR signalling and it has been widely assumed that that the activation of rapamycin-sensitive mTOR plays a critical role in the concomitant hypertrophic response^41^. However, very little is known about how, or if, RE-induced rapamycin-sensitive mTOR signalling regulates downstream process such as protein synthesis, ribosome biogenesis, and mitochondria biogenesis. More importantly, whether rapamycin-sensitive mTOR signalling is necessary for chronic RE-induced hypertrophy has never been investigated. Here we have demonstrated that inhibition of rapamycin-sensitive mTOR signalling via rapamycin injection completely inhibited the induction of ribosome biogenesis by acute RE, but it did not fully prevent acute RE from inducing an increase in c-myc expression or muscle protein synthesis. More importantly, we discovered that rapamycin only partially inhibits the hypertrophy that occurs in response to chronic RE. This is a fundamentally important observation because it indicates that rapamycin-sensitive mTOR-independent mechanisms contribute not only to RE-induced changes in protein synthesis, but also the induction of hypertrophy.

To the best of our knowledge, only three studies have investigated the effects of rapamycin on acute RE-induced changes in protein synthesis. Two of these studies have reported that rapamycin completely inhibited the RE-induced increase in protein synthesis^42^,^43^. However, one study conducted in humans did not demonstrate an effective inhibition of p70S6K phosphorylation^42^; probably due to difficulties in administering rapamycin to humans; moreover, the other study employed a rat squat model that does not induce muscle hypertrophy^44^. In the current study, we used an electrical stimulation-induced RE model, which is known to induce muscle adaptations that mimic those observed with RE in humans^12^,^45^. Our results demonstrate that RE induces an increase muscle protein synthesis and rapamycin only partially blocked this response. Our results are in accordance with those of another recent study in rodents that used electrical stimulation-induced eccentric contractions^13^. In addition, another recent study has reported increases in muscle protein synthesis after endurance exercise in the absence of rapamycin-sensitive mTOR signalling^46^, indicating that both rapamycin-sensitive mTOR-independent and rapamycin-sensitive mTOR-dependent processes regulate muscle protein synthesis following an acute bout of endurance exercise or RE.

A lot of previous studies have identified rapamycin-sensitive mTOR as mTORC1 42,47. However, similar to previous studies^27^,^28^, we observed a decrease in the phosphorylation of Akt Ser473, a marker of mTORC2 signalling, after chronic rapamycin administration. Thus, our observations are consistent with previous studies which have concluded that prolonged rapamycin administration can disrupt signaling by both mTORC1 and mTORC2. Therefore, the rapamycin-sensitive components of RE-induced hypertrophy might be not fully mediated by mTORC1. Moreover, previous studies have never excluded the possibility of unknown forms of rapamycin-sensitive mTOR. Therefore, it is important to consider that the effects of rapamycin may be mediated by more than just the inhibition of mTORC1.

Ribosome biogenesis is currently thought to regulate both muscle protein synthesis and hypertrophy, and it can be regulated by both rapamycin-sensitive mTOR-dependent and rapamycin-sensitive mTOR-independent mechanisms^32^. Previous studies have shown that acute RE increase ribosome biogenesis in both human and rodent skeletal muscles^13^,^36^,^48^. Moreover, chronic RE in human has also shown to induce ribosome biogenesis^49^. In this study, consistent with these studies, we found that acute RE induced ribosome biogenesis and, to our knowledge, we observed for the first time that chronic RE in rodents also induced ribosome biogenesis as well as in human skeletal muscle. Moreover, we determined that rapamycin completely inhibited this effect. Combined, our results indicate that RE induces ribosome biogenesis through an rapamycin-sensitive mTOR-dependent mechanism, and also suggest that ribosome biogenesis is part of the rapamycin-sensitive mTOR-dependent components through which chronic RE induces hypertrophy.

It is known that c-myc regulates protein synthesis and this effect is due, at least in part, to its ability to control the expression genes that regulate ribosome biogenesis^32^,^50^,^51^,^52^. Previous study has shown that mechanical overloading by SA increased association of c-myc at the rDNA promoter^53^. Moreover, recent studies have reported that c-myc-induced ribosomal biogenesis and protein synthesis are, in part, mediated by mTOR signalling^51^,^52^. Previous studies have also shown that RE increases c-myc expression in both human and rodent skeletal muscles^13^,^16^,^35^,^36^,^48^. Similarly, in the present study, we found that c-myc expression was increased after acute RE and this effect was rapamycin-insensitive, which is also in accordance with another study^13^. Therefore, it is possible that the RE induced increase in protein synthesis and subsequent hypertrophy are partially mediated through the rapamycin-sensitive mTOR-independent increase in c-myc expression. However, our results also indicate that the increase in c-myc is not sufficient to induce ribosome biogenesis when rapamycin-sensitive mTOR is inhibited. Thus, the exact role that c-myc plays in RE-induced hypertrophy remains to be defined and this will likely be a fruitful area for future studies.

The mechanisms that regulate c-myc expression in skeletal muscle are relatively unknown. However, similar to Akt phosphorylation (a marker of mTORC2 signalling), c-myc protein expression decreased after chronic rapamycin administration. Interestingly, previous studies have shown that mTORC2 can function as an upstream regulator of c-myc^54^,^55^, suggesting that c-myc expression in skeletal muscles might be regulated by mTORC2. Compared to mTORC1 or rapamycin-sensitive mTOR, the roles of mTORC2 in skeletal muscle are less understood. Nonetheless, previous studies have concluded that mTORC2 can regulate cell growth through a c-myc-dependent pathway^54^,^56^. Combined, these points suggest that the rapamycin-sensitive mTOR-independent increase in protein synthesis by acute RE, and the rapamycin-sensitive mTOR-dependent inhibition of hypertrophy by chronic RE, might be mediated by changes in mTORC2/c-myc signalling.

In summary, one of the most important conclusions that can be drawn from this study is that rapamycin-sensitive mTOR-independent signalling events contribute to the hypertrophic effects of RE. This point raises questions about the possible rapamycin-sensitive mTOR-independent events that might be involved in this process. Obviously these mechanisms remain to be defined, but if future studies are able to define these mechanisms, then they will expose a previously unappreciated, and major component, of the pathway through which RE promotes an increase in muscle mass.

## Methods

### Animal experimental procedures

All experimental procedures in this study were performed in accordance with the guidelines for the Care and Use of Laboratory Animals of Ritsumeikan University and approved by the Ethics Committee for Animal Experiments at Ritsumeikan University. Male Sprague–Dawley rats (~330 g, 11 weeks old) purchased from CLEA Japan (Tokyo, Japan) were housed for 1 week in an environment maintained at 22–24 °C with a 12 h light/dark cycle, with food and water *ad libitum*. After an overnight fast, under isoflurane anesthesia, the right gastrocnemius muscle of the animals was isometrically contracted (ten 3-s stimulation, with a 7-s interval between contraction for 5 sets with 3 min rest intervals) via percutaneous electrical stimulation as previously described^12^, and the left gastrocnemius muscle served as a control. The mTOR inhibitor rapamycin (1.5 mg/kg, 0.25 mg/ml in PBS containing 0.5%DMSO) or placebo (PBS containing 0.5%DMSO) was injected intraperitoneally 1 h before exercise. Muscle samples were obtained 1, 6, and 24 h after a bout of RE, and 48 h after chronic RE (3 times a week for 4 weeks) (n = 5 in each time point). Tissues were rapidly frozen in liquid nitrogen and stored at −80 °C until use.

### Western blotting

Frozen muscle samples were powdered using a beads crusher (μT-12, TAITEC, Saitama, Japan) and 20 mg of powdered samples were homogenized in 10 volumes of homogenization buffer containing 20 mM Tris-HCl (pH 7.5), 1% NP40, 1% sodium deoxycholate, 1 mM EDTA, 1 mM EGTA, 150 mM NaCl, and Halt^TM^ protease and phosphatase inhibitor cocktail (Thermo Fisher Scientific, Waltham, MA). The homogenates were centrifuged at 10,000 × *g* for 10 min at 4 °C. The supernatant was collected, and the protein concentration of each sample was determined using a Protein Assay Rapid kit (WAKO, Osaka, Japan). The samples were diluted in 3 × sample buffer (Cell Signaling Technology, Danvers, MA) and boiled at 95 °C for 5 min. Then, using 5–20% or 10–20% sodium dodecyl sulfate-polyacrylamide gradient gels, equal amounts of protein (20 or 50 μg) were separated using electrophoresis and subsequently transferred to ClearTrans^®^ SP polyvinylidene difluoride membranes (WAKO). After transfer, the membranes were washed in Tris-buffered saline containing 0.1% Tween-20 (TBST) and blocked with RAPIDBLOCK^TM^ SOLUTION (AMRESCO, Solon, OH) for 5 min at room temperature. Membranes were washed and incubated overnight at 4 °C with primary antibody. Antibodies against phospho-Akt (Ser473, cat#9271), total-Akt (cat#9272), phospho-p70S6K (Thr389, cat#9205), total-p70S6K (cat#2708), phospho-rpS6 (Ser240/244, cat#2215), total-rpS6 (cat#2217), phospho-4E-BP1 (Thr37/46, cat#9459), total-4E-BP1 (cat#9452), phospho-ERK1/2 (Thr202/Tyr204, cat#9101), total-ERK1/2 (cat#9102), phospho-p38 MAPK (Thr180/Tyr182, cat#9211), total p38 MAPK (cat#9212), phospho-ULK1 (Ser757, cat#14202), total-ULK1 (cat#8054), LC3 (cat#2775), and c-myc (cat#9402) were obtained from Cell Signaling Technology (Danvers, MA). The UBF (cat#sc-13125) and PGC-1α (cat#516557) antibodies were obtained from Santa Cruz Biotechnology (Santa Cruz, CA) and Millipore (Billerica, MA), respectively. The membranes were then washed again in TBST and incubated for 1 h at room temperature with the appropriate secondary antibody. The membranes were visualized using chemiluminescent reagents, and the bands were detected using the C-DiGit Blot Scanner (LI-COR, Lincoln, NE). The membranes were then stained with Coomassie blue to verify equal loading in all lanes. Band intensities were quantified using Image Studio (LI-COR, Lincoln, NE).

### Muscle protein synthesis

Muscle protein synthesis was measured by the *in vivo* SUnSET method^57^. Under the anaesthesia, 0.04 μmol puromycin/g body wt (Wako, Tokyo, Japan) diluted in a 0.02 M PBS stock solution was injected intraperitoneally, and the gastrocnemius muscle was removed exactly 15 min after puromycin administration. Following homogenization as described above and centrifugation at 2,000 *g* for 3 min at 4 °C, the supernatant was collected and processed for western blotting. A mouse monoclonal anti-puromycin antibody (cat#MABE343, Millipore, Billerica, MA) was used to detect puromycin incorporation, which was evaluated as the sum of intensity of all protein bands in the western blot.

### Real-time PCR

Total RNA was extracted from the powdered muscle sample using ISOGEN II (Nippon Gene, Tokyo, Japan) according to the manufacturer’s instructions. Total RNA concentrations were measured using a Synergy HT (BioTek, Winooski, VT) and 500 ng of total RNA was reverse transcribed into cDNA using a High Capacity cDNA RT kit (Applied Biosystems, Foster City, CA).

Real-time PCR was performed using a TaqMan^®^ Fast Universal PCR Master Mix (Applied Biosystems) and a Mini Optiocon Real-Time PCR System (Bio-Rad, Hercules, CA). TaqMan^®^ Gene Expression Assays (Applied Biosystems) were used to measure the expression of PGC-1α (Rn00580241_m1), c-myc (Rn00561507_m1), β-actin (Rn00667869_m1), GAPDH (Rn01775763_g1), and β2-microgloblin (Rn00560865_m1). In a rodent model of resistance exercise used in this study, GAPDH and β2-microgloblin did not change after RE and rapamycin administration while β-actin significantly increased after RE (Fig. S1). Furthermore, GAPDH was more stable than β2-microgloblin. Therefore, GAPDH was used as the reference gene in this study.

### Ribosomal RNA content analysis

Five microliters of total RNA solution (25 μg muscle/μl TE) was mixed with 1 μl GR Red Loading Buffer (Biocraft, Tokyo, Japan) and 1 μl 100% glycerol, and then loaded on a 1% agarose gel in TBE buffer. The band intensities of 18S and 28S ribosomal RNA (rRNA) were measured using ImageJ software (NIH, Bethesda, MD).

### Myofiber CSA analysis

Mid-belly cross-sections (5 μm thick) were fixed with 10% neutral buffered formalin for 30 min at room temperature, and then sections were stained with hematoxylin and eosin to measure the myofiber cross-sectional area (CSA). The CSA of approximately 300 randomly selected myofibers per muscle were measured by a blinded investigator with ImageJ software (NIH, Bethesda, MD).

### Statistical analyses

Two-way ANOVA (rapamycin × exercise) was used to evaluate changes in the phosphorylated protein, total protein, mRNA, rRNA, muscle wet weight, and muscle fiber CSA. Post-hoc analyses were performed using *t*-tests, with a Benjamini and Hochberg false discovery rate correction for multiple comparisons when significant main effects or interactions were found. Relative changes were compared between groups by paired *t*-test. The level of significance was set at *P* < 0.05.

## Additional Information

**How to cite this article**: Ogasawara, R. *et al*. The role of mTOR signalling in the regulation of skeletal muscle mass in a rodent model of resistance exercise. *Sci. Rep*. **6**, 31142; doi: 10.1038/srep31142 (2016).

## Supplementary Material

Supplementary Information
