# Supplementary material for: The role of mTOR signalling in the regulation of skeletal muscle mass in a rodent model of resistance exercise
Source: Sci Rep. 2016 Aug 9;6:31142. doi: 10.1038/srep31142 (PMC4977552; doi:10.1038/srep31142)
Supplement: Supplementary Information [file srep31142-s1.pdf]

The role of mTOR signalling in the regulation of skeletal muscle mass in a rodent model of resistance exercise

Riki Ogasawara[1,2,*], Satoshi Fujita[3], Troy A. Hornberger[4], Yu Kitaoka[2], Yuhei Makanae[3], Koichi Nakazato[5], Naokata, Ishii[2]

[1]Department of Life Science and Applied Chemistry, Nagoya Institute of Technology, Nagoya, Japan

[2]Department of Life Sciences, The University of Tokyo, Tokyo, Japan

[3]Department of Sport and Health Science, Ritsumeikan University, Kusatsu, Japan

[4]Department of Comparative Biosciences, School of Veterinary Medicine, University of Wisconsin-Madison, Madison, Wisconsin, USA

[5]Department of exercise physiology, Nippon Sport Science University, Tokyo, Japan

[*]ogasawara.riki@nitech.ac.jp

Fig. S1

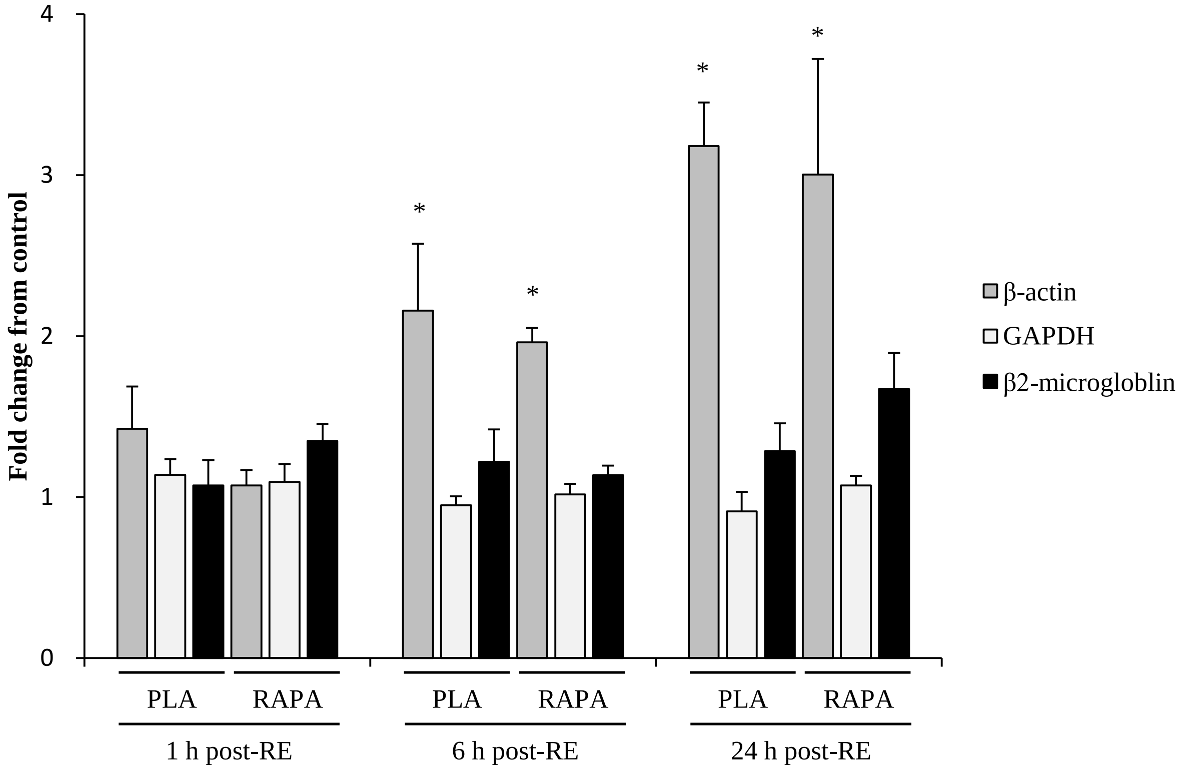

Figure S1. The effect of acute RE and rapamycin administration on mRNA expression of β-actin, GAPDH, and β2-microgloblin. RE , resistance exercise; RAPA, rapamycin; PLA, placebo. Values in the graphs are means + SE. *P<0.05 vs. no exercise control muscle in the same group.

Fig. S2

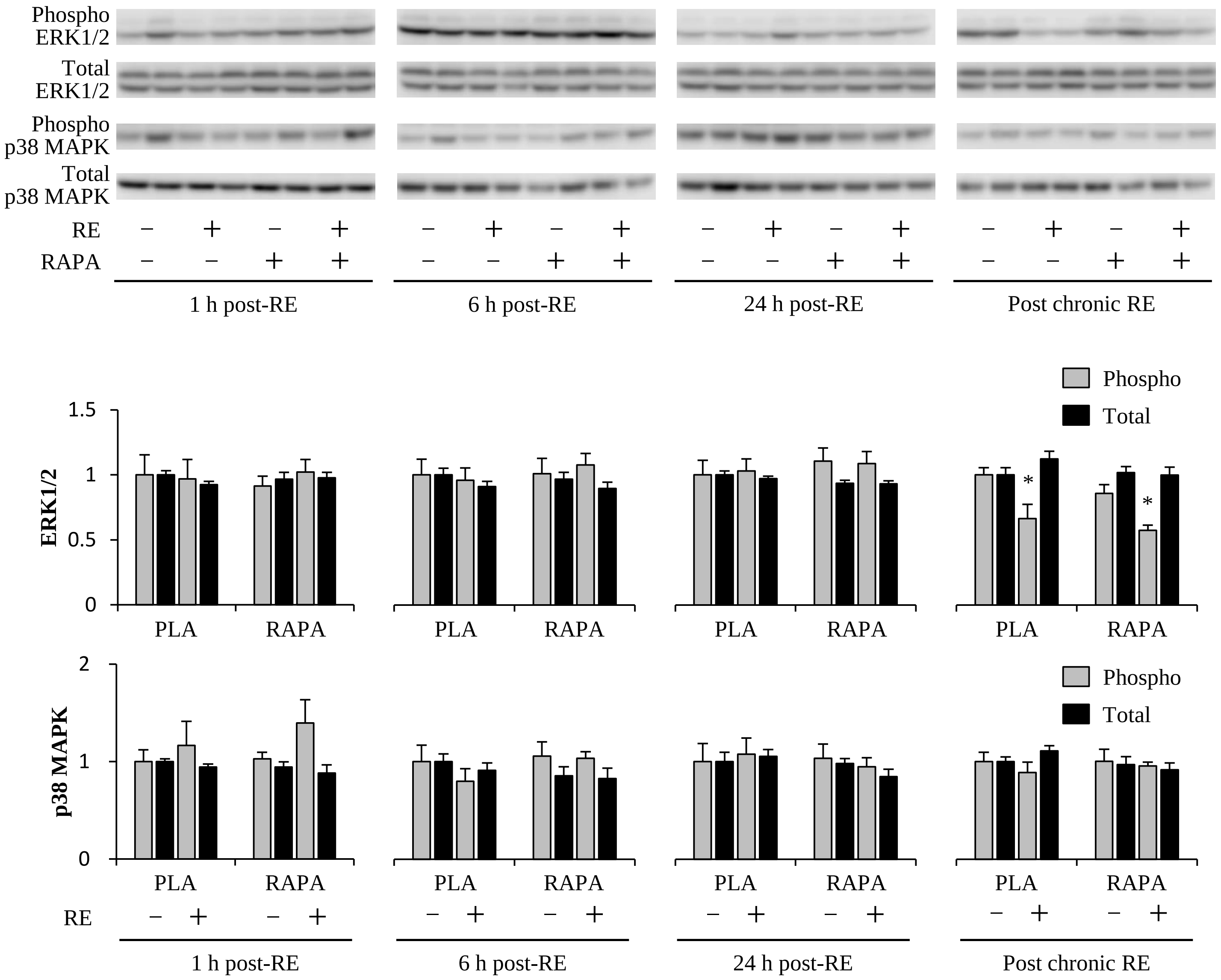

Figure S2. The effect of RE and rapamycin administration on the phosphorylation and total protein content of ERK1/2 and p38 MAPK. RE, resistance exercise; RAPA, rapamycin; PLA, placebo. Values in the graphs are means + SE. **P*<0.05 vs. no exercise control muscle in the same group.
